# Interprofessional Emergency Training Leads to Changes in the Workplace

**DOI:** 10.5811/westjem.2017.11.35275

**Published:** 2017-12-14

**Authors:** Dorothea Eisenmann, Fabian Stroben, Jan D. Gerken, Aristomenis K. Exadaktylos, Mareen Machner, Wolf E. Hautz

**Affiliations:** * Universitätsmedizin Berlin, Department of Anesthesiology and Operative Intensive Care Medicine, Charité, Berlin, Germany; † Universitätsmedizin Berlin, Medical Skills Lab, Charité, Berlin, Germany; ‡ University Hospital Berne, Department of Emergency Medicine, Inselspital, Berne, Switzerland; § Charité Health Acadamy, Berlin, Germany

## Abstract

**Introduction:**

Preventable mistakes occur frequently and can lead to patient harm and death. The emergency department (ED) is notoriously prone to such errors, and evidence suggests that improving teamwork is a key aspect to reduce the rate of error in acute care settings. Only a few strategies are in place to train team skills and communication in interprofessional situations. Our goal was to conceptualize, implement, and evaluate a training module for students of three professions involved in emergency care. The objective was to sensitize participants to barriers for their team skills and communication across professional borders.

**Methods:**

We developed a longitudinal simulation-enhanced training format for interprofessional teams, consisting of final-year medical students, advanced trainees of emergency nursing and student paramedics. The training format consisted of several one-day training modules, which took place twice in 2016 and 2017. Each training module started with an introduction to share one’s roles, professional self-concepts, common misconceptions, and communication barriers. Next, we conducted different simulated cases. Each case consisted of a prehospital section (for paramedics and medical students), a handover (everyone), and an ED section (medical students and emergency nurses). After each training module, we assessed participants’ “Commitment to Change.” In this questionnaire, students were anonymously asked to state up to three changes that they wished to implement as a result of the course, as well as the strength of their commitment to these changes.

**Results:**

In total, 64 of 80 participants (80.0%) made at least one commitment to change after participating in the training modules. The total of 123 commitments was evenly distributed over four emerging categories: *communication*, *behavior*, *knowledge* and *attitude*. Roughly one third of behavior- and attitude-related commitments were directly related to interprofessional topics (e.g., “acknowledge other professions’ work”), and these were equally distributed among professions. At the two-month follow-up, 32 participants (50%) provided written feedback on their original commitments: 57 of 62 (91.9%) commitments were at least partly realized at the follow-up, and only five (8.1%) commitments lacked realization entirely.

**Conclusion:**

A structured simulation-enhanced intervention was successful in promoting change to the practice of emergency care, while training teamwork and communication skills jointly.

## BACKGROUND

Medical error has received considerable attention since the Institute of Medicine estimated that, in the United States alone, as many as 98,000 patients die annually from preventable medical mistakes.^1^ While the exact numbers are disputed, and remain difficult to measure, more recent studies estimate the number of deaths attributable to medical errors to be around 250,000 per year in the U.S.^2^ Emergency departments (ED) are notoriously prone to such errors^3^,^4^, and evidence suggests that one key to decrease the rate of mistakes in acute care settings is to improve teamwork.^5^

Diagnostic accuracy can be increased through interaction in the ED,^6^,^7^ and improved coordination within teams in intensive care is associated with decreased patient mortality.^8^ Transfer of care situations, such as a handover from prehospital to hospital teams, are particularly susceptible to medical errors, due to communication failures and loss of information.^9^ These factors, fortunately, seem to be amenable to training.^10^ The World Health Organization specifically suggested improving interprofessional collaboration as an important way to reduce medical error.^11^ The recent “Call to Action for Emergency Medicine” by Wilbur^12^ highlights the importance of this collaboration, advocating for the implementation of interprofessional education and its evaluation in emergency medicine.

Education is interprofessional “when students from two or more professions learn about, from, and with each other to enable effective collaboration and improve health outcomes.”^12^ One central goal of such education is the improvement of team skills^13^ and communication. Consequently, early educational interventions that improve communication within healthcare teams are likely to be beneficial to patients. Still, professionals who are meant to routinely collaborate with others in an interdisciplinary ED are trained and educated in separate “silos”^12^ in many countries, rendering the development of shared mental models, a common language, or a clear conception of each other’s roles virtually impossible.

## OBJECTIVES

We aimed to conceptualize, implement, and evaluate a training module for final-year medical students, as well as advanced trainees from emergency nursing and student paramedics. The objective was to sensitize participants to professional barriers for their communication across borders, especially in the ED, as it represents an important interface between prehospital and ED teams. Our goal was to establish a mutual understanding of each other’s roles, and of professional self-conceptions. We further aimed to enable participants to conduct basic emergency care for a critically ill patient as an independent interprofessional ad hoc team, with a special focus on communication and team interaction.

## CURRICULAR DESIGN

### Conception

Due to the above-described lack of interprofessional training, we developed a longitudinal, simulation-based training format for three professions based on Kern’s *six-step approach*^14^ (problem identification, needs assessment, formulation of objectives, developing formats, implementation, and evaluation). An interprofessional team consisting of medical, nursing, and paramedic educators planned the educational activity. The resulting training consists of several one-day training modules. We conducted two modules as a pilot. Each module has the same overall structure of an introduction followed by simulated scenarios. The scenarios differ between modules. We invited the same population of student paramedics and emergency nursing trainees, who are both organized in classes. For both groups, the two modules were made a part of their schedule. As a result, most of the student paramedics and emergency nursing trainees participated in both pilot modules and will participate in the following one as a longitudinal course.

Such a longitudinal integration was not possible for medical students, because the training modules were not compulsory and not planned as a longitudinal format, due to difficulties in acquiring a series of time slots in their busy academic schedule. As a result, different medical students participated in the first and second training module. However, this also provided a greater number of medical students the opportunity to attend a training module at least once and relates to real-world circumstances, where teams often form ad hoc without prior acquaintance.^15^

Our long-term objective is to implement this longitudinal format into the new curricula for emergency nursing and paramedics trainees, as well as to offer the format as a voluntary course for medical students. As we have extensive experience with the team-training format in the ED setting,^17^,^18^ we decided to use simulation-enhanced interprofessional education as our educational strategy, due to its well-known positive effects on attitudes towards teamwork and communication.^19^,^20^

### Implementation

After providing oral and written informed consent at the beginning of every module, participants were randomly assigned into four groups, equally staffed with the three professions. Each team met for an introduction session in the morning. The purpose of this first interprofessional meeting was to get acquainted with each other, to discuss each member’s roles, professional self-concepts, common misconceptions, and communication barriers. Every module had a “Topic of the Day”, such as “handover,” “Manchester Triage System,” or “Crisis Resource Management,” which was introduced by an impulse presentation. Furthermore, the interprofessional team of instructors asked for expectations and personal goals for each day.

After introduction, every team rotated through different simulated emergency cases throughout the day. Cases were selected by the interprofessional team of instructors, reflected common emergencies, and contained challenges for interprofessional collaboration, such as team communication and interaction. Every case used high-fidelity simulators or simulated patients. Examples of cases used in the first training module are depicted in Table 1. Each case consists of a prehospital section (for paramedics and medical students), a handover (everyone), and an ED section (medical students and emergency nurses).

After every case, all students underwent extensive structured debriefing by an interprofessional team of instructors, which consisted of members of at least two professions, as well as one expert in communication. Debriefing structure followed the common three-step GAS-model^21^, and is composed of the following parts:

- Gather information from participants (“*How do you feel after this case?*”)
- Analyze information with further questions (“*What went well?;*” “*What happened during handover?;*” “*Do you see any chance for improvement?*”) and directive feedback
- Summarize debriefing with learning goals for the next simulation by the instructor team. Debriefing focuses especially on the “Topic of the Day.”

### Adaption to Participant Feedback

We implemented some changes in our concept after the first training module, based on oral and written feedback from the participants and group discussions among the instructors:

- The number of cases was decreased; simulation/debriefing-time was increased to 90 minutes, allowing for more debriefing time.
- Focus of cases was changed, and more time for paramedic treatment was given: transportation by ambulance was added, so as to balance treatment time between disciplines.
- Due to confusion during debriefing in the first event, roles during debriefing were specified: the medical and communication debriefings were divided between different instructors, so as to establish a more focused observation during simulation. We also allocated a time slot to allow for peer observers to give individual (one-on-one) feedback to their colleagues.

More simulated patients were added, as feedback indicated that they were particularly challenging during scenarios. Furthermore, other challenges, such as pediatric emergencies, distractors, bystanders, and technical incidents, were added to the cases.

## IMPACT/EFFECTIVENESS

Although the relationship between team performance and team culture has long been recognized in acute care,^22^ establishing a link between team characteristics and patient outcome is notoriously difficult.^5^,^23^ One reason for this is that the effect of any educational intervention is likely diluted by the many other factors influencing the transition from individual learning, to behavior within teams, team performance, and finally to patient care, which ultimately determines patient outcome.^5^,^24^ Thus, Cook and West recommend chains of carefully designed studies linking educational interventions to learning effects, learning to behavioral change in the workplace, and behavioral changes to changes in patient care, finally influencing patient outcomes.^24^ Many studies, however, fall short of assessing educational outcomes beyond participant satisfaction. 25,26 “Commitment to Change” (C2C) is one of the few tools that can be used to promote and assess behavioral changes induced by an educational intervention.^27^–^29^ It has been extensively used in different areas, inside and outside healthcare, to stimulate and evaluate performance change.^27^,^29^–^35^ C2C has been associated with behavioral change,^29^,^36^,^37^ and is predictive of success in change initiatives.^34^,^38^ In this C2C approach, participants are anonymously asked to state up to three changes they wish to implement as a result of a course, as well as the strength of their commitment to these changes. After a timespan that allows for implementation, participants are asked to report on their success, and reflect on factors that fostered or hindered implementation.

We translated the original English version of C2C^39^ into German using the established TRAPD (translation, review, adjudication, pre-test, documentation) methodology.^40^ The translated version is available as an appendix.

We collected C2C directly after training (t1), ensuring participant anonymity, while also enabling a follow-up survey after two months (t2). Specifically, we asked participants to generate a unique individual code by appending the first two letters of their mother’s given name, the last two digits of their father’s year of birth, and the first two letters of their place of birth (e.g. PE62BE for a mother named Petra, a father born in 1962, and Berlin as place of birth). For follow-up, we provided the participants with envelopes labeled with their code, containing a follow-up survey on their personal commitments to change. Medical students, who are more difficult to reach as they are not organized into classes, were invited via mail to participate in the follow-up survey. An incentive of 20€ was granted to every medical student participating in follow-up.

We analyzed commitments, together with basic demographic data, in a mixed method approach, both quantitatively and qualitatively. Basic demographic characteristics of our participants and attendance at follow-up are shown in Table 2.

Textual data, such as commitments, or responses regarding factors that fostered or hindered implementation of the intended changes, were inductively categorized by three researchers (DE, FS, and JG) according to Mayring.^41^,^42^ All three researchers (two physicians and one senior medical student) discussed each commitment until full consensus was reached regarding which category was the most appropriate. Emerging categories were defined and adapted, regrouping statements until all commitments were assigned to as little categories as we deemed appropriate. After categorization, the results were presented to an independent psychologist, who was responsible for consistency check and content validation. The process of inductive categorization is often used with qualitative data. The indicators used to assess the quality of qualitative research are generally different from the quantitative methods commonly used in biomedicine, although the quality principles applied to both are similar.^43^

In total, 64 of 90 participants (71.1%) made at least one commitment to change after the training modules (18 trainees of emergency nursing, 22 student paramedics, 15 medical students, and 9 not assignable). That led to a total of 123 commitments made by our participants (see Table 3), which were divided into four broad areas. Commitments were evenly distributed over three emerging categories, namely *communication*, *behavior,* and *knowledge,* as well as a slightly less prominent fourth category, *attitude*. Roughly one third of behavior- and attitude-related commitments refer to interprofessional topics (e.g., “Acknowledge other professions’ work”), and these were equally distributed among professions. Table 3 presents all categories and examples of commitments to change.

At the two months follow-up (t2), 32 participants (50%) provided written comments on their original commitments. At follow-up, 57 of the 62 (91.9%) commitments were reported to be at least partly realized, and only five (8.1%) commitments (still) lacked realization. The best rate of commitment realization was (self-) reported by trainees of emergency nursing, with 13 fully implemented commitments out of 31 (41.9%). We did not observe any significant correlations between the strength of commitments and the probability of their realization (r=0.222; *p*=0.1), suggesting that realization is more strongly influenced by external factors in the workplace than by participant motivation. This hypothesis is further supported by the qualitative analysis of factors that hinder implementation, namely “*not enough practice,”* “*not enough time,”* “*unsupportive colleagues,” and “excessive demand.”* Likewise, the most frequently mentioned factors fostering change were “*practice,” “colleagues,”* and *“teachers.”*

### Validity Argument

In this description of an educational intervention, we report on self-reported commitments to change and self-reported implementation rates. One apparent question that results from the nature of these data is, whether or not C2C is a valid measure of educational outcome for our training. In the following section, we will thus discuss the validity argument for the conclusions drawn from this study, guided by Messick’s five sources of validity evidence as adapted to medical education by Cook et al^44^ and Beckman.^45^

The C2C survey basically consisted of one item: “I commit to complete the following in the next 2 months:” with the option to make up to three statements of anticipated changes, together with a strength of one’s commitment. For all its brevity, this approach has been taken successfully for many years in different contexts.^27^,^29^–^35^ Purkis et al. were able to demonstrate that self-reported intentions of changing behavior were followed by actual behavior changes in physicians following a continuing medical education (CME) intervention.^29^

Content evidence: At present, the use of C2C has rarely been reported in an interprofessional setting. However, behavioral and attitudinal changes were emerging categories in our study and insufficient time was frequently cited as a barrier for realization, consistent with Evan’s findings.^35^ Because there was no possibility to directly measure and observe changes of our participants in their workplace, we chose the well-established method of C2C, which has been developed and validated for this very content.

As for the response process, we report that C2C was part of the evaluation at the end of every module. Statements made by participants were consistent, reflecting a good understanding of the question. All participants had protected time to complete the survey, with an instructor available for questions. We observed a slightly increased motivation to take part in the survey after the second module as some participants already knew the tool and had received their own statements of the first module as a reminder during follow-up. Furthermore, we investigated the possibility of a non-response bias as a possible consequence of the response process. (See below.)

Many participants committed to similar changes, which we were able to cluster into different categories as shown above. Since data from C2C do not allow for elaborate quantitative analyses, we regard this as the best possible internal structure evidence. Due to this relatively new approach in an interprofessional educational setting we, however, fail to provide relationship evidence.

However, regardless of the content of the commitments made or the ability to realize the anticipated changes, the first consequence of the C2C survey was that participants had to reflect on what they had just learned, helping them to identify areas of personal improvement. As a second consequence, at least some participants will try to actually put their committed changes into realization in their workplace (consequences evidence).

### Nonresponse Bias Analysis

Due to the dropout of 50% between first assessment and follow-up, we conducted a nonresponse bias evaluation and tested for differences between the responder and non-responder group using exploratory statistics. Nonresponse bias is a bias resulting from one group of participants being systematically more likely to answer a survey than another;^46^ e.g., participants who were successful in implementing their intended changes could be more willing to report on those successes than participants who could not realize these changes. There were no significant differences between groups in age (p = 0.340; independent samples t-test) and gender (p = 0.294; Fisher’s exact test). However, trainees of emergency nursing (n=14; 77.8% response rate on follow-up) and student paramedics (n=13; 59.1%) were significantly more likely to respond than medical students (n=1; 6.7%; p < 0.01; Pearson’s chi-squared test).

## LIMITATIONS

Relying on self-reports only, our data are inherently limited. Also, although the C2C-approach employed in this study has been extensively used in continuous medical education, further research is warranted to strengthen the link between teaching events, C2C, and objective changes in the workplace. Furthermore, C2C is meant as a tool to enhance change in the workplace, and as such, from a theoretical perspective, its use as a measurement instrument is limited.

Another limitation is the 50% response rate in the follow-up survey, which may introduce nonresponse bias. There is only little reported use of the C2C approach in an interprofessional setting.^35^ Compared to studies surveying students, that report response rates of 46%-31%,^47^–^50^ a 50% response rate in our sample seems satisfactory. However, conclusions about medical students remain limited, despite an incentive, due to the high dropout rate. This effect could be related to poor availability, as the training modules are not part of medical students’ mandatory curriculum, and students hardly participated more than once.

### Lessons Learned and Future Directions

Planning an interprofessional simulation training requires considerable time, coordination, and resources. It is very rewarding to see that the effort has an effect beyond participant satisfaction. C2C is an easy-to-use tool to help students reflect on what lessons to take home – and into their work place. While lack of time is a frequently cited obstacle hindering change,^35^,^51^ a lack of practical training, as well as unsupportive colleagues and teachers in the workplace, seem to be neglected as a factor preventing students from change. As practice is important, interprofessional simulation trainings and internships should be implemented as longitudinal programs in the respective curricula of all involved health professions.

## Supplementary Information



## Figures and Tables

**Table 1:** Student interest in emergency medicine before and after participation in the clinical reasoning elective.

Case (Diagnosis)	Alert & patient presentation	Anticipated course of simulation	IPE Focus
Urinary tract infection and dehydration (SP)	Suspected stroke: geriatric patient with sudden onset of confusion	fast transport into hospital for diagnostics (P)→handover (P→EN/MS)→diagnostics (bloodworks, urine sample, cCT scan) organizing transfer to ICU (EN+MS)	Good handover needed according to high risk of information loss on a patient who can’t give information himself.
Minor head injury (SP)	Bicycle accident: drunk and uncooperative patient with laceration on forehead and bruised right arm	Wound management, immobilization and transport (P)→handover (P→EN/MS) → examination and decision on further diagnostics (EN+MS)	Developing a common concept of managing an uncooperative patient out of different strategies.
Hypoglycaemia and leg injury (Simulator)	Unclear coma: unconscious patient with leg injury is found by joggers in a park setting near a tree	Treatment hypoglycemia, wound and pain management, transport (P/MS) → handover (P/MS → EN/MS) → neurological examination, blood works, x-ray leg and prioritization of further treatment (EN/MS)	Gathering and transferring information of an unknown patient and an unclear course of events.
Acute coronary syndrome (Simulator)	Transfer transport I: Patient in the ER of a smaller hospital with STEMI to be transferred to the next hospital with cardiac catheter	Patient goes into cardiac arrest (Ventricular Fibrillation) during handover (EN/MS → P/MS) → immediate Advanced Life Support → ROSC after 3 shocks and first drug administration	Switching to resuscitation immediately especially in a situation of unclear leadership during hand over.
Esophageal variceal bleeding with hemorrhagic shock (Simulator)	Transfer transport II: Patient after liver transplantation to be transferred from ICU to a different hospital	planned transfer of a postoperative patient → patient spits blood and goes into hemorrhagic shock during handover (EN/MS → P/MS) → Managing circulatory problem (infusion/transfusion), securing airway and initiating further treatment	Managing an unforeseen situation in mixed teams.

*IPE*, Interprofessional Education; *SP*, simulated patient; *P*, paramedic student; *EN*, emergency nursing trainee; *MS*, last year medical student; *ER*, emergency room; *cCT*, cranial computer tomography; *ICU*, intensive care unit; *STEMI*, ST-elevation myocardial infarction; *ROSC*, return of spontaneous circulation.

**Table 2:** Demographic characteristics of participants of first and second training module by professional and participation status (age measured in years).

	Emergency nursing			Paramedics			Medical students			Total*		
				
	Participated	Initial C2C	Follow-up	Participated	Initial C2C	Follow-up	Participated	Initial C2C	Follow-up	Participated	Initial C2C	Follow-up
1^st^ Training model												
No. (% female)	15 (78.6)	10 (80.0)	7 (85.7)	20 (40. 0)	11 (45.5)	5 (40.0)	11 (36.4)	9 (33.3)	0 (-)	49 (46.9)	33 (48.5)	12 (66.7)
Mean age (SD)	33.53 (7.89)	31.50 (6.87)	32.43 (7.79)	21.79 (3.05)	21.82 (3.03)	20.40 (1.14)	27.82 (3.97)	27.22 (3.80)	- (-)	27.18 (7.32)	26.67 (6.23)	27.42 (8.48)
2^nd^ Training model												
No. (% female)	11 (72.7)	8 (75.0)	7 (71.4)	14 (28.6)	11 (36.4)	8 (37.5)	7 (42.9)	6 (33.3)	1 (0.0)	41 (41.5)	31 (45.2)	20 (50.0)
Mean age (SD)	29.20 (4.19)	30.57 (4.32)	30.83 (4.67)	22.86 (3.48)	23.45 (3.73)	22.75 (3.69)	26.29 (1.98)	26.50 (2.07)	23.00 (-)	27.00 (6.84)	27.92 (7.33)	28.53 (8.94)

*C2C,* commitment to change; *SD*, standard deviation; *No*., Number.

* Differences to preceding columns result from participants unassignable to their professional group.

**Table 3:** Categorization of “commitment to change” statements from first and second training module.

Category	Examples of quotes	Emergency nursing (n=26)	Paramedic (n=34)	Medical students (n=18)	Not assignable (n=11)	Total
Knowledge	“revise cardiology”, “revise ABCDE scheme”, “consolidate basics”	6	7	12	5	30 (24.4%)
Communication	“greet the paramedic team”, “clear and structured handover”, “targeted communication”, “attentive listening”	16	11	8	7	42 (34.3%)
Behavior/teamwork	“appreciate other professions, get to know them personally”, “10 seconds for 10 minutes principle”	7	7	9	1	24(19.5%)
Attitude/others	“improve understanding for other professions”, “appreciation”, “respect”, „become more confident“, “reduce coffee consumption”	10	8	2	7	27 (22.0%)
Total		39 (31.7%)	33 (26.8%)	31 (25.2%)	20 (16.2%)	123
