# Supplementary material for: Interprofessional Emergency Training Leads to Changes in the Workplace
Source: West J Emerg Med. 2017 Dec 14;19(1):185–92. doi: 10.5811/westjem.2017.11.35275 (PMC5785192; doi:10.5811/westjem.2017.11.35275)
Supplement: Supplementary file 1 [file wjem-19-185-s001.docx]

**🡺 Selbstverpflichtung zur Veränderung**

**„commitment to change“**

Teilnehmer ID

Bitte gib im Folgenden eine oder mehr Veränderungen an, die Du Dir in Anbetracht der heutigen Lernaktivität (Interprofessionelle Simulation) vornimmst.

Gib so viele Veränderungen an, wie Dir geeignet scheinen.

Bitte beachte dabei, dass diese in den nächsten ein bis zwei Monaten abgeschlossen werden können.

Ich verpflichte mich in den nächsten zwei Monaten Folgendes zu erfüllen:

***Selbstverpflichtung zur Veränderung (commitment to change) Nummer 1***

____________________________________________________________________________________________________________________________________________________________________________________________________________________________________________________________________________________________________________________________________

Wie stark ist Deine Absicht, die oben genannte Veränderung vor zu nehmen. (bitte kreuze an)

| ① | ② | ③ | ④ | ⑤ |
| --- | --- | --- | --- | --- |
| Niedrigste Ebene der Selbstverpflichtung |  |  |  | Höchste Ebene der  Selbstverpflichtung |

Ich verpflichte mich in den nächsten zwei Monaten Folgendes zu erfüllen:

***Selbstverpflichtung zur Veränderung (commitment to change) Nummer 2***

____________________________________________________________________________________________________________________________________________________________________________________________________________________________________________________________________________________________________________________________________

Wie stark ist Deine Absicht, die oben genannte Veränderung zu machen. (bitte kreuze an)

| ① | ② | ③ | ④ | ⑤ |
| --- | --- | --- | --- | --- |
| Niedrigste Ebene der Selbstverpflichtung |  |  |  | Höchste Ebene der  Selbstverpflichtung |

Ich verpflichte mich in den nächsten zwei Monaten Folgendes zu erfüllen

***Selbstverpflichtung zur Veränderung (commitment to change) Nummer 3***

____________________________________________________________________________________________________________________________________________________________________________________________________________________________________________________________________________________________________________________________________

Wie stark ist Deine Absicht, die oben genannte Veränderung zu machen. (bitte kreuze an)

| ① | ② | ③ | ④ | ⑤ |
| --- | --- | --- | --- | --- |
| Niedrigste Ebene der Selbstverpflichtung |  |  |  | Höchste Ebene der  Selbstverpflichtung |

Bitte schreib Dir die drei Punkte noch einmal ab oder mach Dir ein Foto, wir werden in ein paar Wochen nachfragen☺.

***Die Angaben sind streng anonym, die einzelnen Punkte können keinen Personen zugeordnet werden.***

***Wir danken Dir sehr für Deine Hilfe und hoffen, dass Du einen tollen lehrreichen Tag hattest.***
